# Magnetic anisotropy and phononic properties of two-dimensional ferromagnetic Fe_3_GeS_2_ monolayer

**DOI:** 10.1016/j.isci.2024.110781

**Published:** 2024-08-22

**Authors:** Yu Wei, Hui Liu, Ke Wang

**Affiliations:** 1 Xi’an University of Posts & Telecommunications, Shaanxi 710121, China

**Keywords:** Physics, Magnetism

## Abstract

In 2023, Fe_3_GeS_2_ monolayer with Curie temperature of 630 K is predicted, which is promising to be used in next-generation spintronic devices. However, its magnetic anisotropy and phononic properties are still unclear. In this paper, we implemented the first-principles calculations on Fe_3_GeS_2_ monolayer, and found its ferromagnetic ground state with robustness to the −1.5%–1.3% biaxial strain. Meanwhile, the out-of-plane magnetic anisotropy dominated by dipolar interaction is found in Fe_3_GeS_2_ monolayer. Finally, we studied the phononic properties to identify the dynamical stability of Fe_3_GeS_2_ monolayer and highlight the contribution from the anharmonic interaction of optical phonons to the thermal expansion coefficient. We also find two single-phonon modes can be used to design quantum mechanical resonators with a wide cool-temperature range. These results can provide a comprehensive understanding of the magnetism and phonon properties of two-dimensional (2D) Fe_3_GeS_2_, beneficial for the application of 2D Fe_3_GeS_2_ in spintronics.

## Highlights

• The ground state of Fe_3_GeS_2_ monolayer is robust to the −1.5% to 1.3% biaxial strain
• There are two single-phonon modes (9.08 THz and 11.12 THz) in the phonon spectrum
• The out-of-plane magnetic anisotropy is dominated by dipolar interaction
• The contribution from optical phonons to thermal expansion coefficient is significant

## Graphical abstract

**Figure:**
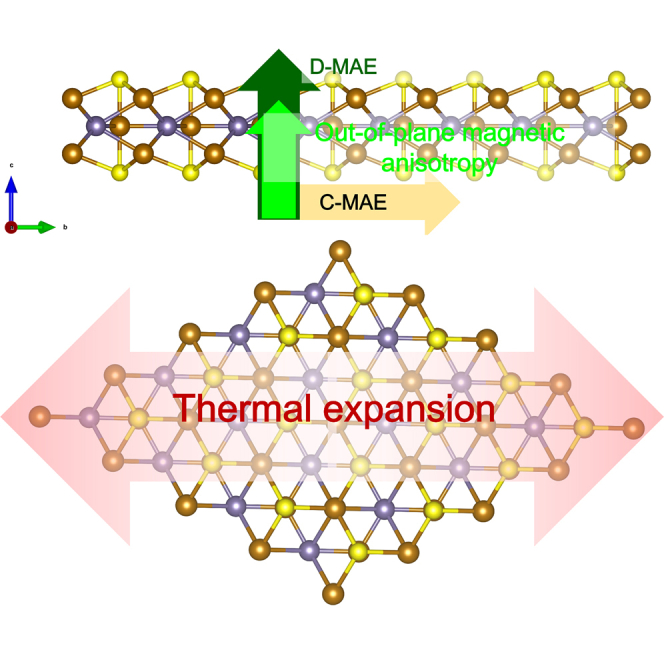

## Introduction

The successful exfoliation of two-dimensional (2D) graphene has opened up substantial research topics on the fascinating properties and potential applications of 2D materials. In the initial stage of studying 2D materials, researchers focused on black phosphorene, transition metal dichalcogenides (TMDs), hexagonal boron nitride, etc., all of which are nonmagnetic (NM). In 2017, the 2D ferromagnetic (FM) CrGeTe_3_ and CrI_3_ were mechanically exfoliated from the bulk crystals by Gong et al. and Huang et al.*,*^1^^,^^2^ which shed light on the applications of 2D materials in spintronics. Subsequently, a large of 2D magnets are predicted and prepared, including CrCl_3_,^3^ Fe_3_GeTe_2_,^4^ CrTe_2_,^5^ MnBi_2_Te_4_^6^ with nontrivial topology, Weyl half-semimetal PtCl_3_,^7^ and antiferromagnetic (AFM) MnPS_3_^8^ and MXenes.^9^^,^^10^ In recent years, these 2D magnets attracted extensive research attention in the field of materials science and condensed matter physics, due to their interesting properties, such as large spin Seebeck coefficient,^11^ controllable magnetoresistance,^12^ and quantized anomalous Hall effect.^13^ These intriguing properties render 2D magnets promising candidates in fabricating spintronic devices. Currently, spintronics devices have been widely applied in the fields of signal transfer,^14^ data storage,^15^ biomedicine,^16^ and energy conversion.^17^

However, a fatal flaw of 2D magnets is their weak magnetic stability and low magnetic phase-transition temperature (Curie temperature for FM; Néel temperature for AFM), which hinders strongly their application in spintronics. For instance, the Curie/Néel temperatures are ∼60 K for CrGeTe_3_^1^, ∼45 K for CrI_3_^2^, and 89 K for FePS_3_,^18^ which is much lower than the room temperature. Therefore, in recent years, researchers have devoted themselves to exploring methods to improve the magnetic phase-transition temperature of 2D magnetic materials or design novel 2D magnetic materials with high Curie/Néel temperature. At present, it is recognized that the out-of-plane magnetic anisotropy is key for 2D magnets to break the Mermin-Wagner theorem and to resist thermal fluctuation. The relationship between Curie/Néel temperature and the out-of-plane magnetic anisotropy can be written as:

(Equation 1) $$T_{c}=-\left(ZJ_{lf}+ZK_{lf}+A_{\text{zz}}\right)S_{0}\cdot \frac{1}{3K_{B}}\text{,}$$

where *J*_*lf*_, *K*_*lf*_, and *A*_*zz*_ are the isotropic magnetic exchange coupling coefficient, out-of-plane magnetic anisotropic parameter, and out-of-plane single-point anisotropic parameter, respectively. *K*_*B*_ is the Boltzmann constant, while *S*_0_ is the altitude of spin vector on each magnetic lattice. The details of Equation 1 can be referred to a study by Wang et al.^19^ Hence, many strategies have been employed to enhance the out-of-plane magnetic anisotropy and elevate the Curie/Néel temperature of 2D magnets, such as strain engineering,^20^^,^^21^ charge doping,^22^^,^^23^ surface functionalization,^24^^,^^25^ atomic doping,^26^^,^^27^ intercalation,^28^^,^^29^^,^^30^ and external manipulation.^31^^,^^32^ Meantime, researchers also predicted many new 2D magnets with high Curie/Néel temperatures.^33^^,^^34^^,^^35^ For example, Wen et al.^36^ prepared 2D FM CuCr_2_Te_4_ flakes with thickness-dependent Curie temperature (260 K–320 K) by the heteroepitaxial growth. Based on Fe_3_GeTe_2_ monolayer with the Curie temperature of 150 K,^37^ Yang et al.^38^ predicted 60 types of easy exfoliable and highly stable magnetic A_3_BX_2_ monolayers by machine learning and high-throughput computation. Among these 60 types of A_3_BX_2_ monolayer, Fe_3_GeS_2_ monolayer with excellent dynamical and thermal stability owns the highest Curie temperature of 630 K, much higher than room temperature 300 K. Therefore, if it can be successfully prepared in time, it will bring revolutionary progress to spintronic devices based on 2D magnets, making 2D spintronic devices have great application potential in quantum communication, data storage, biomedicine, and other fields. However, in their study, the Curie temperature was estimated using Monte Carlo simulation based on the magnetic exchange coupling, without exploring the magnetic anisotropy in Fe_3_GeS_2_ monolayer.

In this paper, we would explore the magnetic anisotropy in Fe_3_GeS_2_ monolayer by the first-principles calculations. Besides, in the spintronic devices fabricated by the 2D magnets, the performance and reliability of the devices heavily depend on the thermal conduct and thermal expansion of 2D magnets. Phononic properties of 2D magnets are the basis for analyzing and exploring the thermal conduct and thermal expansion. Therefore, we would also delve into the phononic properties and thermal expansion of Fe_3_GeS_2_ monolayer, laying a theoretical foundation for its application in 2D spintronic devices. We found the out-of-plane magnetic anisotropy dominated by dipolar interaction in Fe_3_GeS_2_ monolayer, robust to the −1.5%–1.3% biaxial strain. Meantime, we also find two single-phonon modes with frequencies of 9.08 THz and 11.12 THz, which can be used to design quantum mechanical resonators with wide cool-temperature range. Besides, we also calculate the Grüneisen constants of all phonons and thermal expansion coefficient (TEC) of FM Fe_3_GeS_2_ monolayer, highlighting the contribution from the anharmonic interaction of optical phonons to the TEC.

### Computational details

**Figure 1:**
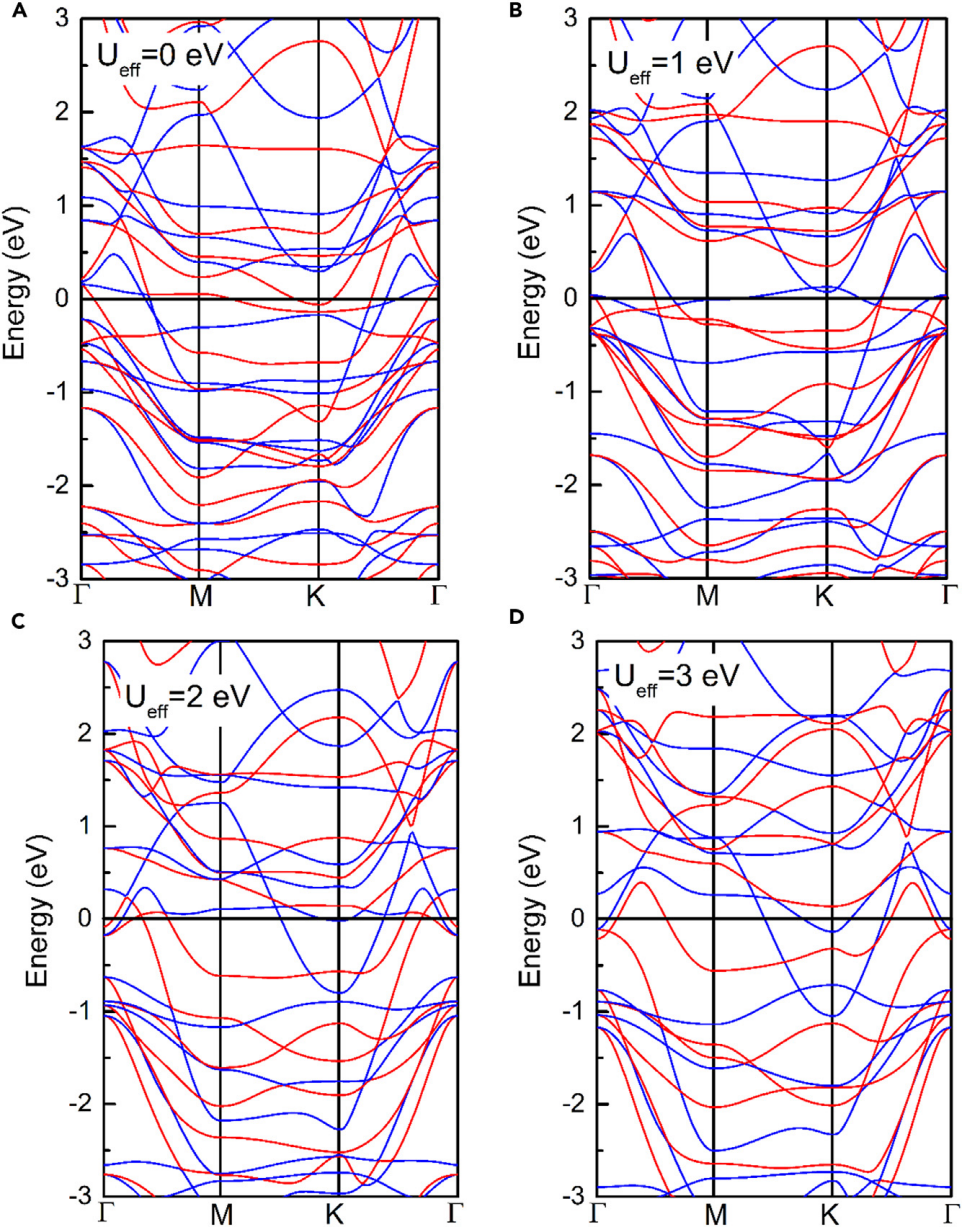
DS-PAW calculated band structures of Fe_3_GeS_2_ monolayer with different “*U*” values The used "*U_eff_*" values are 0 eV (A), 1 eV (B), 2 eV (C), and 3 eV (D), respectively.

In our simulations, the calculations of structural relaxation, electronic density of states, and phonon dispersion were obtained by Device Studio program,^39^ which provides several functions for crystal visualization, modeling and simulation. In this paper, all these calculations were implemented by DS-PAW software integrated in Device Studio program.^40^ In DS-PAW software, the projected augmented wave (PAW) method was employed to describe the coupling between atomic nuclei and extra-nuclear electrons, while we selected the Perdew-Burke-Ernzerhof (PBE) method of general gradient approximation (GGA) as exchange-correlation functional.^41^^,^^42^ To suppress the non-physical interaction between adjacent layers, a 15 Å vacuum space was imposed along the out-of-plane direction. The convergence limits of energy and force were set as 10^−7^ eV and 0.001 eV/Å in the relaxation of geometrical structure with cutoff energy of 500 eV and 5×5×1 Monkhorst-Pack (MP) grid. The phonon dispersions were calculated using an MP grid of 9×9×1 for a 2×2×1 supercell, based on the density function perturbation theory (DFPT).^43^ Besides, it has been reported in previous studies^44^^,^^45^^,^^46^ that including the Hubbard “*U*” into the PBE functional will overestimate the lattice parameters and magnetic moment of based-Fe_3_GeTe_2_ materials seriously. Meantime, we calculated the band structures of Fe_3_GeS_2_ monolayer with different “*U*” values, as shown in Figure 1. It can be observed that the metallic state of Fe_3_GeS_2_ monolayer is robust to the value of Hubbard “*U*”. Therefore, the “*U*” parameter was not included in our calculations.

## Results and discussion

**Figure 2:**
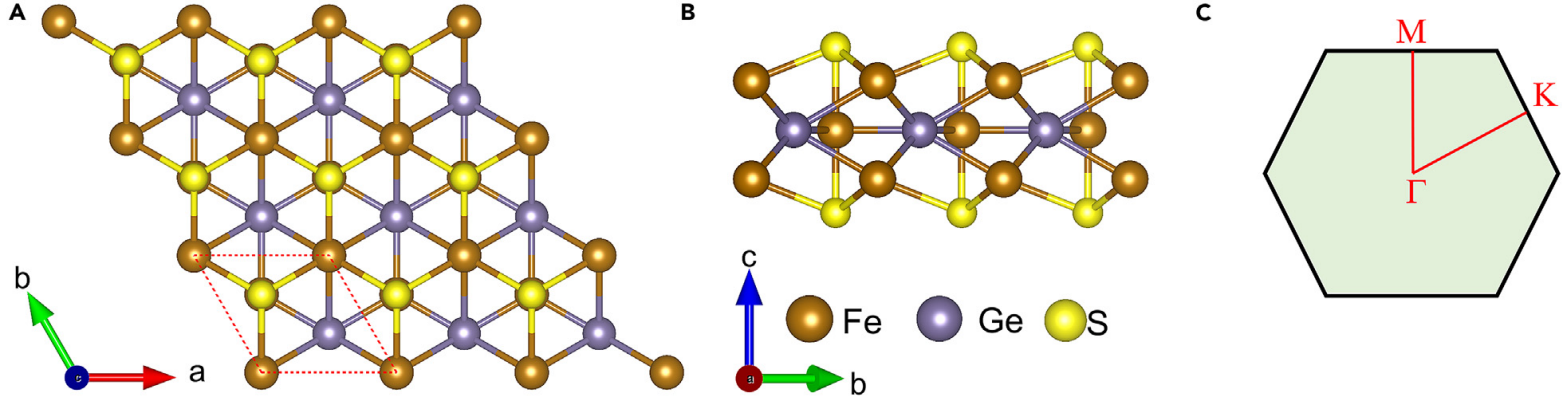
Geometrical structure and high-symmetry path of Fe_3_GeS_2_ monolayer (A and B) are the top and side views of geometrical structure, and (C) is the high-symmetry path in the irreducible Brillouin zone. In (A) and (B), the Fe, Ge, and S atoms are represented by the brown, grayish purple and gold balls, respectively.

**Figure 3:**
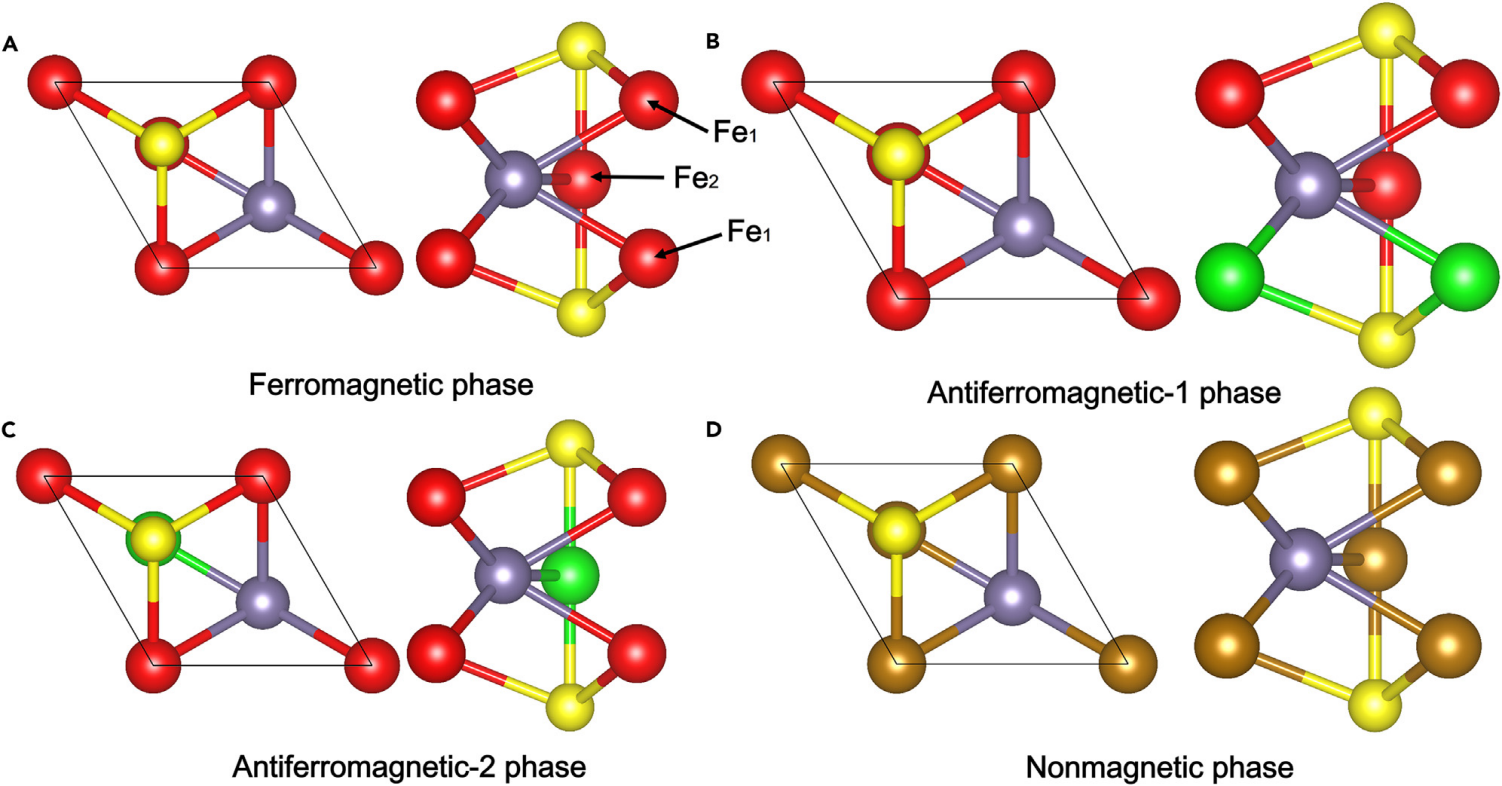
Four possible magnetic phases in the Fe_3_GeS_2_ unit cell Four possible magnetic phases include ferromagnetic (FM) (A), antiferromagnetic-1 (AFM-1) (B), antiferromagnetic-2 (AFM-2) (C), and nonmagnetic (NM) (D) phases.

**Figure 4:**
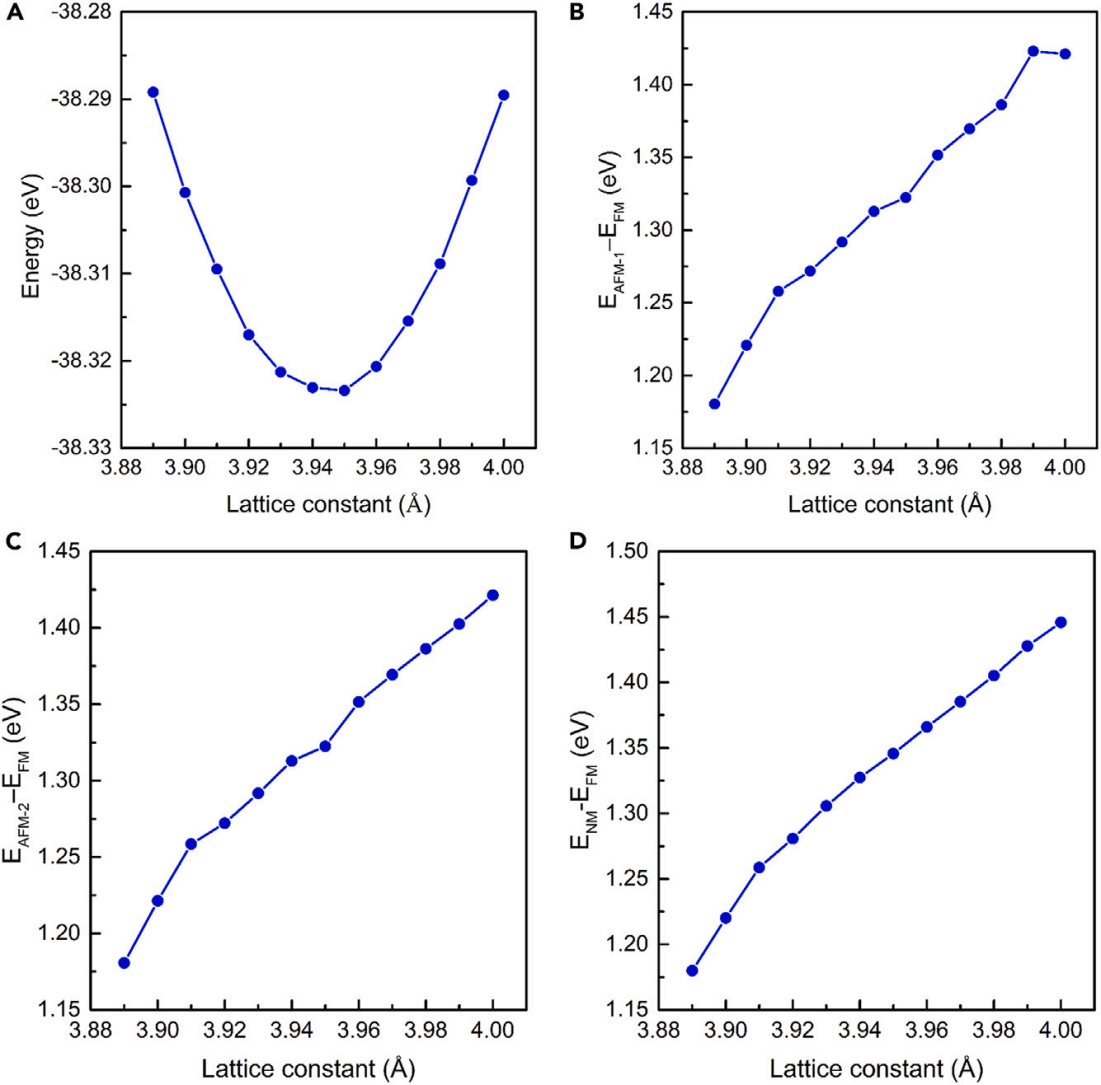
The curve of energy at possible magnetic phases versus strain (A) is the energy of Fe_3_GeS_2_ monolayer at FM phase, while (B–D) are the energy differences between FM and, AFM-1, AMF-2 and NM phases obtained by DS-PAW, respectively.

In this paper, the model of crystal Fe_3_GeS_2_ is established by using the bulk Fe_3_GeTe_2_ as a template. The bulk Fe_3_GeS_2_ is hexagonal, with the space group of *P*6_3_/*mmc* (No. 194). The Fe_3_GeS_2_ monolayer is exfoliated from the bulk Fe_3_GeS_2_, and its space group is *P*-6*m*2 (No. 187). The top and side views of Fe_3_GeS_2_ monolayer are shown in Figures 2A and 2B, where the Fe, Ge, and S atoms are represented by the brown, grayish purple and gold balls, respectively. In Fe_3_GeS_2_ monolayer, there are five sub-layers, where Fe_3_Ge substructure is sandwiched by two S layers. The red dashed line marks the unit cell of Fe_3_GeS_2_ monolayer in Figure 2A. In this unit cell, there are six atoms, including three Fe atoms, one Ge atom, and two S atoms. In the unit cell of Fe_3_GeS_2_ monolayer, these three Fe atoms can be divided in two inequivalent types Fe_1_ (including the top and bottom Fe atoms) and Fe_2_, based on their positions and the crystal symmetry. Hence, there are four possible magnetic phases in the unit cell of Fe_3_GeS_2_ monolayer, including FM, AFM-1, AFM-2, and NM magnetic configurations, as shown in Figure 3. When both the spins of two Fe_1_ atoms are parallel to that of Fe_2_ atom, it results in an FM phase, as shown in Figure 3A. In AFM-1 phase, the spin of top Fe_1_ is antiparallel to the bottom Fe_1_, but is parallel to that of Fe_2_. If the coupling between Fe_2_ and two Fe_1_ atoms is AFM, while the coupling between two Fe_1_ atoms is FM, it leads to AFM-2 phase. To determine the magnetic ground state, we calculated the energies of Fe_3_GeS_2_ monolayer at these four possible magnetic phases, as shown in Figure 4. We can find the Fe_3_GeS_2_ monolayer with FM phase owns the lowest energy at lattice constant of 3.95 Å. In Figures 3C and 3D, it can be found that the energies of AFM-1, AFM-2 and NM phases are ∼1.322 eV, ∼1.323 eV, and ∼1.346 eV higher than the FM phase as lattice constant is of 3.95 Å. Meantime, with the lattice constant increasing, these energy differences also elevate significantly. When the lattice constant is smaller than 3.95 Å, all of the energy differences between AFM-1, AFM-2, NM, and FM phases are still positive, indicating the energy of FM phase is lowest among these four possible magnetic phases. According to the least-energy principle, it can be drawn that the magnetic ground state of Fe_3_GeS_2_ monolayer is FM, and the optimized lattice constant is *a* = *b* = 3.95 Å. Furthermore, this FM magnetic ground state of Fe_3_GeS_2_ monolayer is robust to the biaxial strain within the range of −1.5%–1.3%.

**Figure 5:**
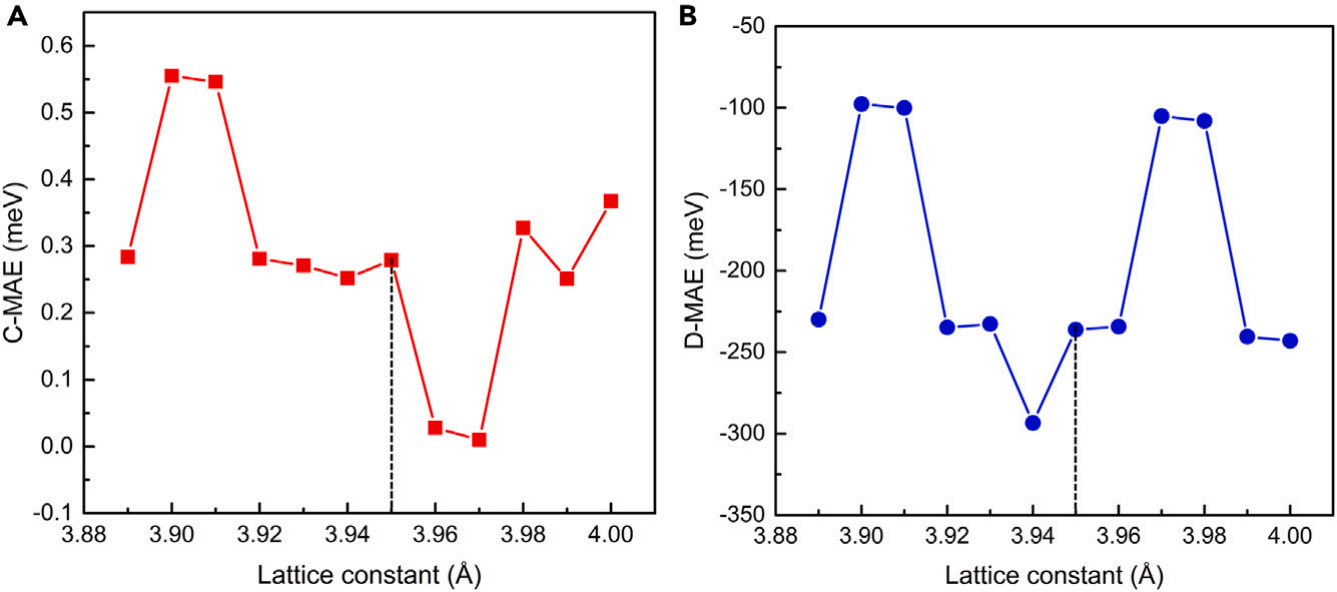
Magnetic anisotropy energy of Fe_3_GeS_2_ monolayer with strain The magnetocrystalline anisotropic energy (C-MAE) (A) and magnetic dipole anisotropic energy (D-MAE) (B) of Fe_3_GeS_2_ monolayer obtained by DS-PAW.

For 2D magnets, magnetic anisotropy is essential to resist thermal fluctuation and keep the long-range magnetic order. Magnetic anisotropy, introduced by the spin-orbit coupling, is composed of magnetocrystalline anisotropic energy (C-MAE) and dipolar magnetic anisotropic energy (D-MAE). In this paper, C-MAE is calculated by the XXZ model^47^:

(Equation 2) $$E_{C-\text{MAE}}=E_{\text{ZZ}}-E_{\text{XX}}\text{,}$$

where the E_XX_ (E_ZZ_) is the energy of 2D magnet with the spin along *x*- (*z*-) direction. In the XXZ model, the energy with spin along the *x*-direction is considered equal to the spin along the *y*-direction, resulting in-plane magnetic isotropy. Thus, the C-MAE only considers the energy difference of 2D magnets as the spin is along *x*- and *z*-directions. The dipolar interaction energy in magnet can be calculated by^48^:

(Equation 3) $$E^{d}=\frac{\mu_{0}{\left(g\mu_{B}\right)}^{2}}{2}\sum_{i\neq j}\frac{{\left|R_{ij}\right|}^{2}S_{i}\cdot S_{j}-3\left(R_{ij}\cdot S_{i}\right)\left(R_{ij}\cdot S_{j}\right)}{{\left|R_{ij}\right|}^{5}}\text{,}$$

where *g* of 2 is the gyromagnetic ratio, *μ*_*B*_ and *μ*_0_ are the Bohr magnon and the vacuum permeability, respectively. **R**_*ij*_ is the position vector between the *i*- and *j*-magnetic lattices, while the spin angular momentum of the *i*- (*j*-) magnetic lattice is ***S***_*i*_ (***S***_*j*_). According to Equation 3, it can be found that the dipolar interaction energy relies on the relative position of spin pair and their spin orientations. Early in 2002, Politi et al.^49^ reported the dipolar coupling between single-domain FM particles could induce long-range FM order. Based on the strong and tunable dipole-dipole interaction, Young et al.^50^ realized fast two-qubit entangling gates, providing significant speedups for quantum algorithms. Meantime, Utesov^51^ proposed that dipolar forces originating from the dipole-dipole interaction can lead to biaxial anisotropy in the reciprocal space of antiferromagnets with skyrmion, and the dipolar forces is considered as the critical ingredient to stabilize the nanometer-sized skyrmions in antiferromagnets.^52^ Hence, it is recognized that dipolar forces can result in the complicated sequences of magnetic phase transition in magnets under an external magnetic field.^53^^,^^54^ To obtain the D-MAE, we make a difference in the dipole interaction energy of spin angular momentum along the *z*- and *x*-directions. The obtained magnetic anisotropic energies are plotted in Figure 5. Interestingly, the C-MAE keeps positive revealing an in-plane magnetocrystalline anisotropy. At the optimized lattice constant (3.95 Å), the C-MAE is 0.28 meV. When the lattice constant is stretched to 3.97 Å, the C-MAE of Fe_3_GeS_2_ monolayer decreases to 0.01 meV indicating a nearly isotropic magnetism. As the lattice constant is compressed to 3.90 Å, the C-MAE increases to 0.56 meV, suggesting an enhancement of in-plane magnetocrystalline anisotropy. For D-MAE, it keeps negative, revealing an out-of-plane magnetic dipolar anisotropy. At the optimized lattice constant (3.95 Å), the D-MAE is −236.19 meV, while the sum of C-MAE and D-MAE is −235.91 meV, revealing an out-of-plane magnetic anisotropy in the optimized Fe_3_GeS_2_ monolayer. Furthermore, the D-MAE is within the range of −293.50∼-97.68 meV, with the lattice constant changing from 3.89 to 4.00 Å. These results suggest the Fe_3_GeS_2_ monolayer owns out-of-plane magnetic anisotropy dominated by the dipolar interaction, and this out-of-plane magnetic anisotropy is robust to the biaxial strain within the range of −1.5%–1.3%. It is worth emphasizing that this out-of-plane magnetic anisotropy dominated by the dipolar interaction is rare in previous synthesized 2D ferromagnets, such CrGeTe_3_, CrI_3_, Fe_3_GeTe_2_, which is promising to realize dipolar-induced magnon chirality.

**Figure 6:**
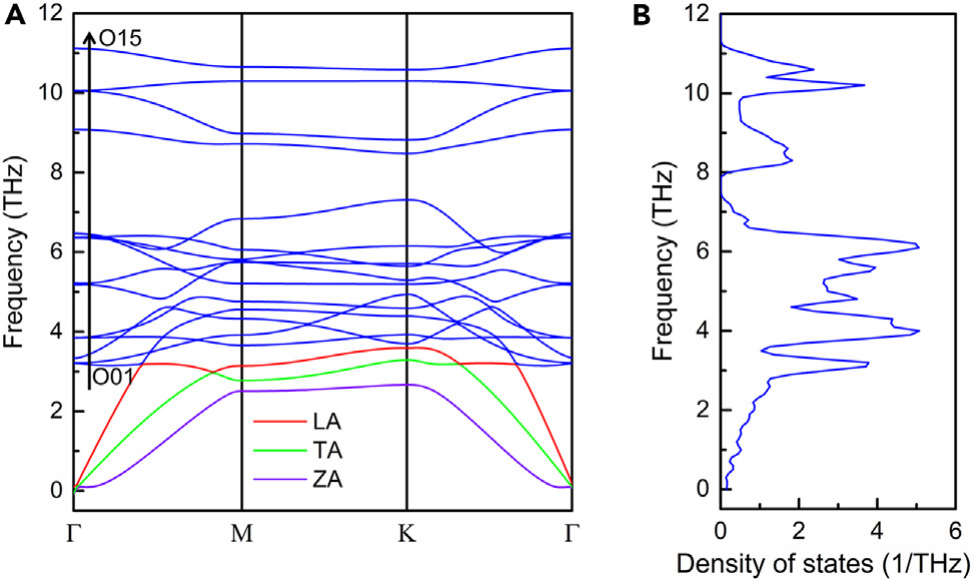
The phonon dispersion and phonon density of states of Fe_3_GeS_2_ monolayer obtained by DS-PAW (A) is phonon dispersion, while (B) shows the phonon density of states.

**Figure 7:**
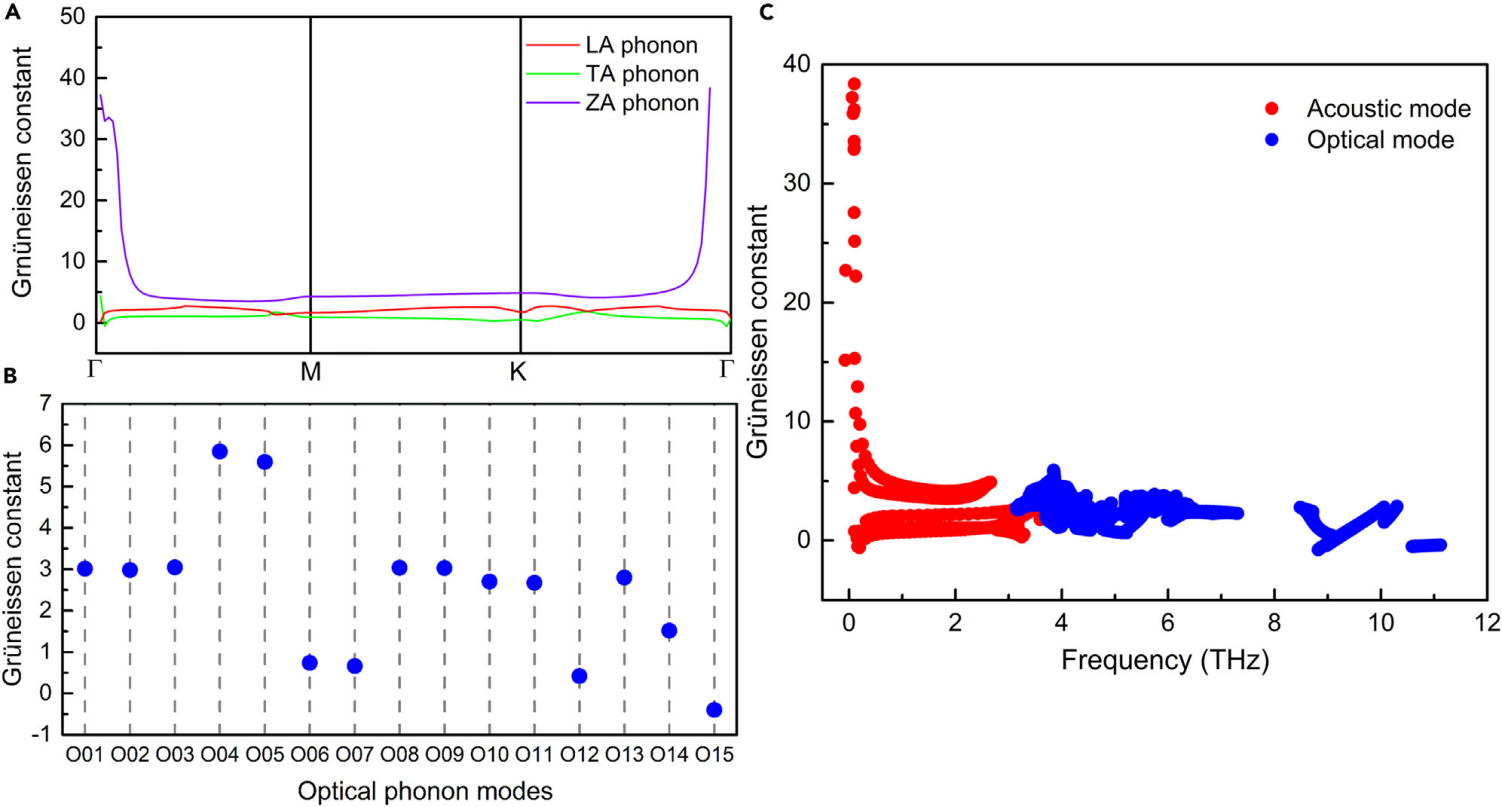
Grüneisen constants of phonons (A) is Grüneisen constants of acoustic phonons along the high-symmetry path, while (B and C) are Grüneisen constants for fifteen optical phonons at the center of Brillouin zone and all phonons, respectively.

To identify the dynamic stability of optimized Fe_3_GeS_2_ monolayer, we calculated its phonon dispersion based on the DFPT, shown in Figure 6. There is little imaginary frequency in the phonon dispersion and phonon density of states (PDOS), suggesting the dynamic stability of our optimized Fe_3_GeS_2_ monolayer. In phonon dispersion, there are 18 phonon branches including 3 acoustic branches and 15 optical branches, because there are six atoms in the unit cell of Fe_3_GeS_2_ monolayer. These three acoustic branches are named as longitudinal acoustic (LA), transverse acoustic (TA), out-of-plane acoustic (ZA) phonons, and their eigenvectors describe the translation of the Fe_3_GeS_2_ monolayer along *x*-, *y*-, and *z*-directions, respectively. It has been reported that the thermal conductivity usually is dominated by the anharmonic interaction of acoustic phonons,^55^^,^^56^ which is fundament for the performance and reliability of the 2D devices. We mark the LA, TA, and ZA branches by the red, green, and purple solid lines in phonon dispersion, as shown in Figure 6A. To estimate the anharmonicity of acoustic phonons in Fe_3_GeS_2_ monolayer, we calculated the Grüneisen constant (γ_***q****,s*_) by^57^:

(Equation 4) $$\gamma_{q,s}=-\frac{a}{2\omega_{q,s}}\cdot \frac{d\omega_{q,s}}{da}\text{,}$$

where *ω*_***q****,s*_ is the phonon frequency of the *s*-branch at the wave vector of ***q***, and *a* is the lattice constant. In this paper, the Grüneisen constant was calculated by applying a 2% biaxial strain to lattice at 0 K, and the obtained Grüneisen constants of acoustic phonons are presented in Figure 7A. As known, a larger absolute value of Grüneisen constant suggests a stronger anharmonicity. In Figure 6A, the absolute Grüneisen constant of ZA phonon is larger than LA and TA phonons, indicating the strong anharmonic interaction with other phonon modes. Meantime, the Grüneisen constant of ZA remains positive with the wave vector changing, suggesting a softening phonon frequency of ZA mode with lattice expansion. At the long-wavelength limit (near Γ point), the Grüneisen constants of LA and TA phonons are 0.18 and 4.42, respectively, which is much smaller than ZA phonon (37.24). These results are different from other 2D materials where ZA phonon owns remarkable negative Grüneisen constant, such as graphene,^58^ biphenylene,^59^ and black phosphorene.^60^ As the wave vector increases, the Grüneisen constant of LA phonon also remains positive, but there is a negative Grüneisen constant (−0.51) of TA phonon.

Compared with acoustic phonons, optical phonon modes can be used to measure thermal conductivity,^8^ evaluate the thickness of 2D materials,^61^ and design quantum mechanical resonator.^62^ Here, we also investigate and analyze the optical phonons in Fe_3_GeS_2_ monolayer. In the phonon dispersion of Fe_3_GeS_2_ monolayer, there are fifteen optical phonons named from O01 to O15, as shown in Figure 6A. We can find two sing-phonon optical modes O12 and O15 with frequencies of 9.08 THz and 11.12 THz, respectively, which is rare in other 2D materials, because PDOS always is composed of the mixed contribution from coupled phonon modes. In sing-phonon optical mode, there is no degeneracy, rendering it promising to design quantum mechanical resonators. In designing quantum mechanical resonators, how to cool the resonator to its ground state is a critical problem. Generally, the cooling temperature *T*_cool_ should be smaller than $hf/k_{B}$,^63^ and *h*, *f*, *k*_*B*_ are Planck’s constant, phonon frequency, and Boltzmann’s constant, respectively. For O12 and O15 modes, the corresponding maximum cooling temperatures *T*_cool_ are 432.63 K and 529.83 K, higher than room temperature and can be realized by standard cryogenic methods. Meantime, the Grüneisen constants of fifteen optical phonons at the center of Brillouin zone (Γpoint) were also calculated by Equation 4, as shown in Figure 7B. Obviously, the O15 mode owns a negative Grüneisen constant of −0.4, suggesting a frequency softening with lattice expansion. The largest Grüneisen constant of 5.85 occurs at O04 mode, but is still smaller than the ZA phonon at the long-wavelength limit.

**Figure 8:**
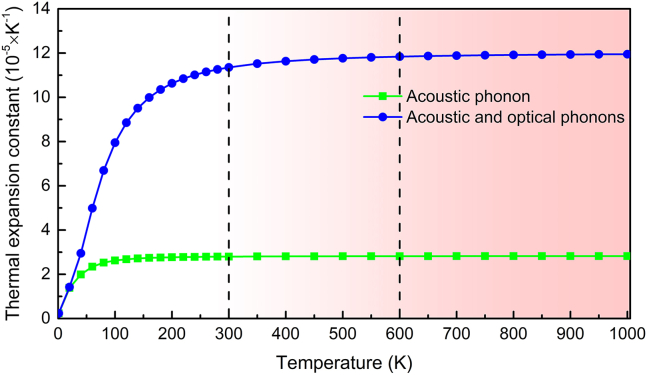
The calculated thermal expansion coefficient (TEC) of Fe_3_GeS_2_ monolayer

In 2D devices, the difference in TEC between the substrates and 2D material can induce strain inevitably, and this strain increases with the temperature. At high temperature, this induced strain could destroy the geometrical structure of 2D devices, leading to the performance degradation of 2D devices. Based on these Grüneisen constants, the TEC *α* can be calculated by^64^:

(Equation 5) $$\alpha =\frac{k_{B}}{V_{0}\cdot B_{2D}}\sum_{q,s}\gamma_{q,s}\frac{{\left(\text{h}\omega_{q,s}/k_{B}T\right)}^{2}\;\exp\left(\text{h}\omega_{q,s}/k_{B}T\right)}{{\left(\exp\left(\text{h}\omega_{q,s}/k_{B}T\right)-1\right)}^{2}}\text{,}$$

where *V*_0_ and *B*_2*D*_ are the volume of Fe_3_GeS_2_ primitive cell and the bulk modulus of Fe_3_GeS_2_ monolayer. The bulk modulus of Fe_3_GeS_2_ monolayer is calculated as 2,165.67 N/m by using the Yang’s modulus and Poisson ratio in a study by Long and Yang.^38^ The calculated TEC of Fe_3_GeS_2_ monolayer is plotted in Figure 8. At 20 K, the TEC contributed by acoustic phonons is 1.39 × 10^−5^ 1/K, which is 97.89% of that considering the contribution from both acoustic and optical phonons. As temperature increases to 300 K, the TEC by only considering the contribution from acoustic phonons is 2.80 × 10^−5^ 1/K. Meantime, the TEC by considering the contribution from both acoustic and optical phonons is up to 11.84 × 10^−5^ 1/K whose 76.35% comes from the optical phonons. Obviously, the contribution from optical phonons grows remarkably with temperature, because the Grüneisen constants of optical phonons are comparable to acoustic phonons, as shown in Figure 7C. With temperature increasing, more and more optical phonons are excited, and then the anharmonic interaction between optical phonons becomes strengthening. Therefore, the contribution from optical phonons to TEC of Fe_3_GeS_2_ monolayer is enhanced significantly, which has also been observed in SiP_2_ monolayer.^65^

### Conclusions

According to first-principles calculations implemented in Device Studio program, we find the magnetic ground state of Fe_3_GeS_2_ monolayer is the FM, and this FM state is robust to the −1.5%–1.3% biaxial strain. Meantime, we also find the out-of-plane magnetic anisotropy is dominated by dipolar interaction in Fe_3_GeS_2_ monolayer. This out-of-plane magnetic anisotropy is beneficial for Fe_3_GeS_2_ monolayer to resist thermal fluctuation and sustain long-range FM order. In phonon dispersion, there are two single-phonon modes with frequencies of 9.08 THz and 11.12 THz, which can be used to design quantum mechanical resonator with a wide cool temperature range. Besides, the Grüneisen constants of all phonons and TEC of FM Fe_3_GeS_2_ monolayer were calculated, which highlights the contribution from the anharmonic interaction of optical phonons to the TEC. Our study provides theoretical support for the application of FM Fe_3_GeS_2_ monolayer in future spintronic devices.

### Limitations of the study

This study implemented first-principles calculations by Device Studio program to investigate the magnetic anisotropy and phononic properties of ideal 2D FM Fe_3_GeS_2_ monolayer with high Curie temperature of 630 K, and found the out-of-plane magnetic anisotropy dominated by dipolar interaction, two single-phonon modes with frequencies of 9.08 THz and 11.12 THz, and the large contribution from optical phonons to thermal expansion.

Nonetheless, this study is not without its limitations that warrant further attention. Generally, the first-principles calculation and Monte Carlo simulation often overestimate the magnetic phase-transition temperature of 2D magnets, underscoring the necessity for additional experimental corroboration. The largest limitation of the study is that 2D FM Fe_3_GeS_2_ has not been successfully prepared, and experimental corroboration of its interesting properties cannot be carried out. This limitation presents a pivotal direction for future research endeavors on 2D FM Fe_3_GeS_2_.

## Resource availability

### Lead contact

Further information and requests for resources and reagents should be directed to and will be fulfilled by the lead contact, Ke Wang (kewang@xupt.edu.cn).

### Materials availability

This study did not generate new unique materials.

### Data and code availability

• Data reported in this paper will be shared by the lead contact upon request.
• This paper does not report original codes.
• Any additional information required to reanalyze the data reported in this paper is available from the lead contact upon request.

## Acknowledgments

Y.W. acknowledges the 10.13039/501100007128Natural Science Foundation of Shaanxi Province (2024JC-YBMS-325). K.W. acknowledges the support from 10.13039/501100001809National Natural Science Foundation of China (NNSFC) (12204373). We gratefully acknowledge HZWTECH for providing computation facilities.

## Author contributions

Conceptualization, Y.W. and H.L.; methodology, Y.W. and K.W.; investigation, Y.W. and K.W.; software, K.W.; writing – original draft, Y.W.; writing – review and editing, K.W. and H.L.; funding acquisition and project administration, Y.W. and K.W.

## Declaration of interests

The authors declare no competing interests.

## STAR★Methods

### Key resources table

**Table:** 

REAGENT or RESOURCE	SOURCE	IDENTIFIER
**Software and algorithms**		

Device Studio (DS-PAW) 2022	HONGZHIWEI TECHNOLOGY	https://cloud.hzwtech.com/web/product-service?id=6

### Experimental model and study participant details

Our study does not use experimental models typical in the life sciences.

### Method details

The detailed numerical setting in first-principles calculations have been presented in computational details.

### Quantification and statistical analysis

Our study does not include statistical analysis or quantification.
